# Analyzing the impact of team-building interventions on team cohesion in sports teams: a meta-analysis study

**DOI:** 10.3389/fpsyg.2024.1353944

**Published:** 2024-03-15

**Authors:** Sang Hyun Kwon

**Affiliations:** Department of Physical Education, Yonsei University, Seoul, Republic of Korea

**Keywords:** group cohesion, group-based intervention, interactive sports, meta-analysis, teambuilding

## Abstract

**Introduction:**

Participation in team sports requires collaboration among multiple individuals over an extended period. Success in the game relies on more than just individual excellence; it necessitates effective teamwork. Team-building interventions have been shown to enhance team functioning, particularly in fostering cohesion among sports teams. This study aims to identify crucial factors in team-building interventions that contribute to improved team cohesion in sports teams.

**Methods:**

A comprehensive meta-analysis of 15 articles was conducted to identify the crucial factors in team-building interventions that contribute to improved team cohesion in sports teams. The analysis focused on the age of participants, level of performance, and duration of interventions.

**Results:**

The results of the analysis revealed that the positive impact of team-building was found to be most pronounced when the participants were between 15 and 20 years old, performed at collegiate teams, and engaged in interventions lasting more than 2 weeks. Among the four types of cohesion in sports teams, individual attraction to the group task (ATG-T) emerged as the aspect most influenced by team-building interventions.

**Discussion:**

These findings provide valuable insights into the factors influencing the success of team-building interventions in enhancing team cohesion within sports teams.

## 1 Introduction

Psychological interventions in sports have proven effective in enhancing athletes’ skill development, team cohesion, and team performance. Among these interventions, team-building has emerged as a prominent strategy for promoting effective collaboration among team members, thereby strengthening cohesion and team performance in sports teams. This approach has been employed to optimize the functionality of sports teams, resulting in improved team performance.

This study aims to explore the impact of team-building interventions on cohesion within sports teams. While numerous investigations have reported favorable effects of team-building on team cohesion (Cogan and Petrie, 1995; Prapavessis et al., 1996; Stevens and Bloom, 2003; Senécal et al., 2008; Kim and Kim, 2012; Durdubas and Koruc, 2023; Tassi et al., 2023), it remains challenging to assert that team-building interventions yield effective results. Some studies, such as those by Bloom and Stevens (2002), Kilty (2000), Kwon (2022), Prapavessis et al. (1996), and Rainey and Schweickert (1988), did not report positive developments in group cohesion.

Moreover, improvements in cohesion achieved through team-building interventions were sometimes transient, with studies indicating that cohesion levels were not sustained throughout the season (Cogan and Petrie, 1995; Stevens and Bloom, 2003). Drawing definitive findings about the effectiveness of team-building in sports is complicated by the diversity of methods and designs employed in these interventions, which yield unexpected results and necessitate an integrated examination of previous studies.

In the meta-analysis conducted by Carron et al. (2002), the impact of team-building on four subgroups of cohesion – GI-T (group integration–task), GI-S (group integration–social), ATG-T (individual attractions to the group-task), and ATG-S (individual attractions to the group-social) – was examined, with reported effect sizes of 0.471, 0.349, 0.676, and 0.463. Martin et al. (2009) conducted a meta-analysis on team-building interventions within sports teams, reporting an effect size of 0.427. Their analysis revealed that team-building interventions had the most substantial impact on cognitions (*g* = 0.799), with goal setting as the exclusive method coming in second (*g* = 0.714). The effect sizes of task and social cohesion were 0.263 and 0.214.

While team-building is known to have a positive effect on team cohesion, in actual application, its implementation time is limited. Therefore, to ensure that the cohesion effect is evident in sports teams, understanding the factors that should be considered in team-building interventions is crucial. This study seeks to determine which moderator variables such as gender, age, athletes’ level, group size, and intervention duration, enhance the effect and which factors do not need to be considered.

## 2 Methodology

This methodology conforms to the relevant guidelines of the Preferred Reporting Items of Systematic Reviews and Meta-Analyses (PRISMA) Statement and ensures that the necessary scientific information is provided in the field (Page et al., 2021).

### 2.1 Study selection and inclusion criteria

For this meta-analysis, literature selection focused on research studies examining the effectiveness of team-building interventions in interactive sport teams. The selection process followed rigorous and systematic procedures, incorporating keyword searches in computerized databases and employing a snowball sampling approach.

The computer-based search covered various databases, including PsychINFO, PsycARTICLES, SPORT Discus, and Google Scholar. This comprehensive search strategy involved using a range of keywords, such as “team-building in sport,” “team-building intervention in sport,” “team-building and cohesion,” and various combinations.

Two independent reviewers extracted the following data from each article: study design, total number of participants, gender, age, intervention duration, and athletes’ skill level. The accuracy of the extracted or calculated data was verified by comparing the data collection forms of the two investigators.

### 2.2 Dependent variables: cohesion

Team-building in sports teams can yield various outcomes, including enhanced cohesion. Carron and Spink (1993) developed a conceptual framework for team-building interventions in sports teams, designating group cohesiveness as the primary result of this process. Within this framework, four subgroups of cohesion, specifically GI-T, GI-S, ATG-T, and ATG-S, serve as dependent variables when assessing the impact of team-building interventions (Eys and Kim, 2017).

### 2.3 Moderating factors

#### 2.3.1 Gender

Within the studies under review, two distinct demographic cohorts were examined. Specifically, 5 studies with 38 cases focused on male participants, while 10 studies with 14 cases centered around female participants.

#### 2.3.2 Age of participants

The participants’ ages were divided into three groups: under the age of 15, 15–20 years old, and over 20. Specifically, two studies with eight cases focused on participants under 15, while nine studies with 29 cases targeted the 15–20 age group, and four studies with 15 cases focused on participants over the age of 20.

#### 2.3.3 Sample size

The sample size was categorized into three groups: under 20 participants, 20–30 participants, and over 30 participants. More specifically, five studies with 17 cases were centered on under 20 participants, while another five studies with 18 cases were aimed at the 20–30 participants group. Additionally, five studies with 17 cases were focused on participants comprising over 30 participants.

#### 2.3.4 Skill level

The analysis covered a range of team proficiency levels. High school and collegiate teams were each represented in five studies with 16 effect sizes, whereas professional club teams were featured in five studies with 20 effect sizes.

#### 2.3.5 Length of intervention

This study also investigated the duration of a team-building intervention as a potential moderator for their effectiveness. The intervention durations were classified into three groups: less than 2 weeks, 2 to 20 weeks, and 20 weeks or more. There were 8 studies with 32 cases that fell within the 2 to 20 weeks category, while 6 studies with 18 cases had intervention lasting over 20 weeks. Additionally, one study with two cases had an intervention duration of less than 2 weeks.

### 2.4 Coding methodology

Following established norms for meta-analytic research, we meticulously designed our coding procedure to thoughtfully capture and quantify crucial study characteristics and outcomes. Our comprehensive coding approach involved systematically extracting 11 essential pieces of information from each study. This included details such as authorship, year of publication, study setting, study design type, sport type, duration of intervention, athletes’ skill level, gender of participants, number of participants in experimental and control groups, means and standard deviations of intervention effectiveness at pretest and posttest, as well as effect size or measures of effectiveness.

### 2.5 Effect size calculations

The computation of effect sizes was conducted using R-4.3.2 for Windows.^1^ This program provides various options for calculating effect sizes, and we chose Hedges g (Hedges and Olkin, 2014), an effect size adjusted to consider differences in sample size and sample variance. In interpreting the magnitude of effect sizes, we followed Cohen’s (1988) guidelines. Specifically, a Hedges g of 0.80 was considered a large effect size, 0.50 signified a medium effect size, and 0.20 indicated a small effect size.

## 3 Results

### 3.1 Study selection

**Figure 1:**
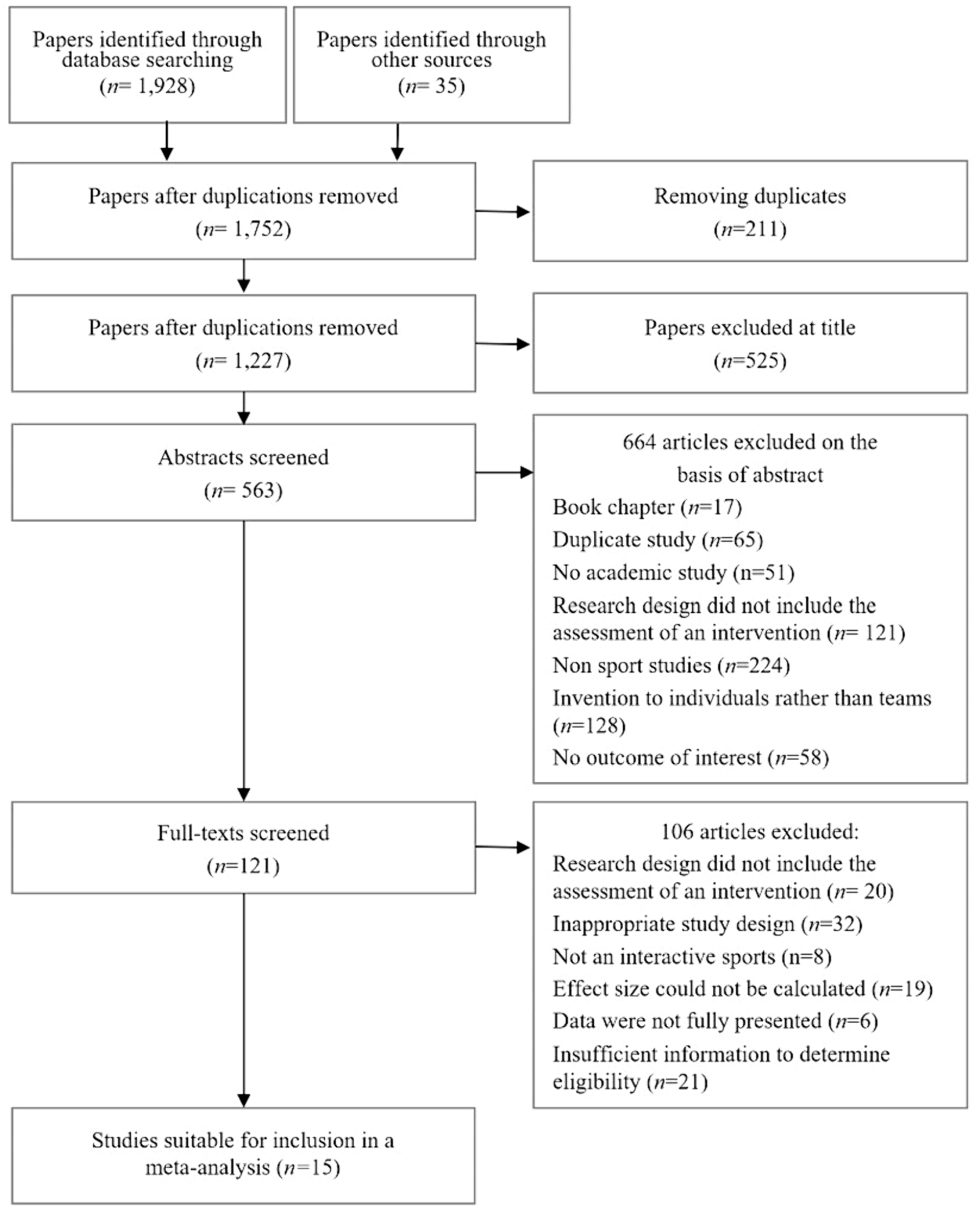
Flowchart of the systematic review process according to the PRISMA protocol declarations.

Following a database search, a total of 1,928 documents were initially identified, with 35 documents found through snowballing methods. After removing duplicates, 1,752 articles remained. Subsequently, 525 articles were excluded based on title screening. Application of the inclusion criteria led to the exclusion of an additional 664 articles. This left us with 121 articles that underwent full-text screening, focusing on articles potentially relevant to the impact of team-building interventions on cohesion in sports teams. To ensure methodological rigor, studies lacking the necessary statistical information for calculating effect sizes were excluded from the meta-analysis. Following these criteria, a total of 15 studies, comprising 52 cases, were considered eligible for inclusion in the meta-analysis (refer to Figure 1 for details).

### 3.2 Assessment of risk of bias

To assess the risk of bias in the included articles, we used the Cochrane Risk of Bias Tool (Higgins and Altman, 2008). This tool assesses each article based on a checklist comprising five items: randomization process, deviation from the intended intervention, missing outcome data, measurement of the outcome, and selection of the reported result. We then categorized each article’s overall bias risk as low risk (indicating low risk across all items), some concerns, and high risk (indicating high risk of bias in at least one domain). Low risk indicates better methodological quality, while high risk suggests a high risk of bias.

**Figure 2:**
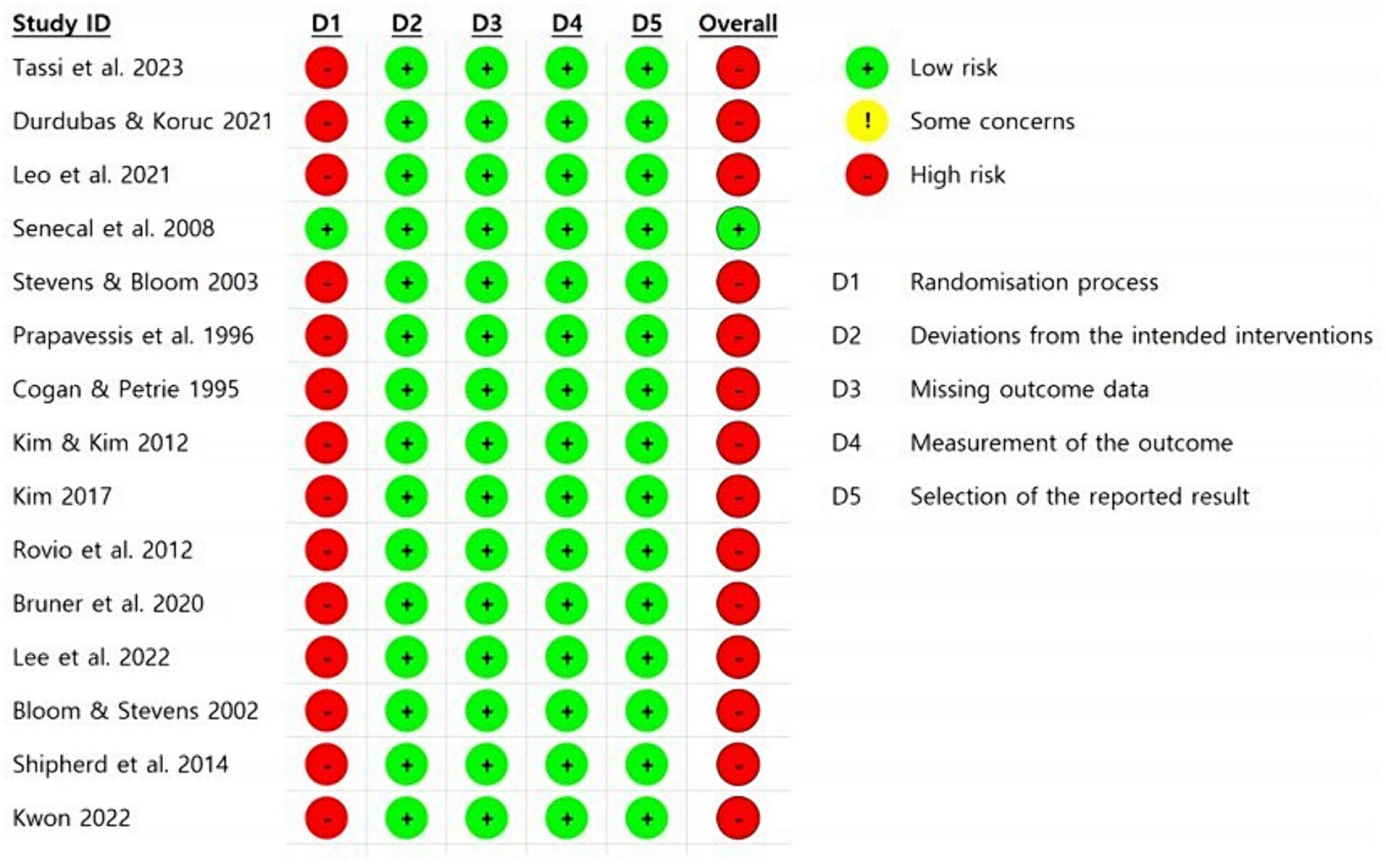
Assessment of risk of bias in the included studies.

Figure 2 provides a visual representation of risk of bias evaluations for each domain of the Cochrane Risk of Bias tool. Out of all included articles, 1 article (6.7%) had a low overall risk of bias, while 14 articles (93.3%) exhibited a high overall risk of bias. However, except for the randomization process domain, the other four checklist items showed low risk across all 15 articles.

The high prevalence of ‘high’ risk is attributed to the inherent challenges in randomly selecting teams, particularly in studies involving interactive sports teams. This difficulty arises from the complexities associated with randomly assigning teams in research focused on sports team dynamics.

### 3.3 Overall analysis

#### 3.3.1 Overall effect size

**Figure 3:**
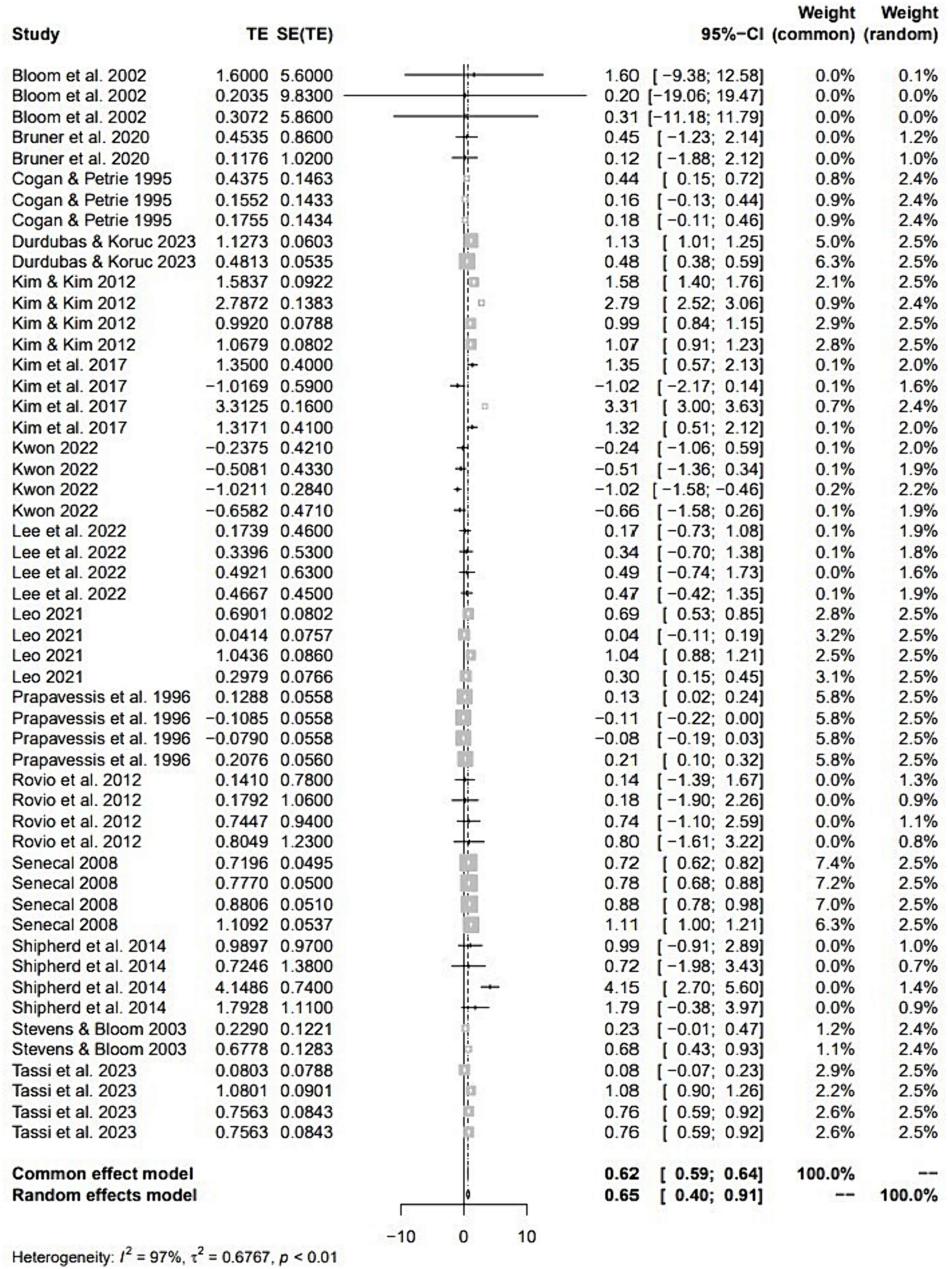
Forest plot of meta-analysis for team building intervention on cohesion in sports teams. The individual effect sizes are identified as Hedges g with lower and upper limits of 95% CIs.

The meta-analysis results, drawn from 52 individual cases extracted from 15 papers, are presented in Table 1. The table covers both the overall analysis and outcomes related to five moderating variables influencing cohesion. Additionally, Figure 3 illustrates a forest plot depicting effect sizes for the 52 individual cases. The overall analysis of these cases showed a significant moderate effect size (ES = 0.65, 95% CI = [0.40; 0.91]) of team-building intervention on cohesion. Additionally, the I^2^ heterogeneity statistic indicated a significant level of heterogeneity at 96.9%.

**Table 1:** Effect sizes of dependent variables.

Dependent variable	*n*	*Effect size*	*I*^2^ (%)	95% CI	
				LL	UL
Overall	52	0.65	96.9	0.39	0.91
GI-T	14	0.56	96.8	0.23	0.89
GI-S	15	0.52	96.7	0.01	1.02
ATG-T	11	1.06	97.9	0.17	1.95
ATG-S	12	0.56	96.6	0.22	0.91

#### 3.3.2 Publication bias

**Figure 4:**
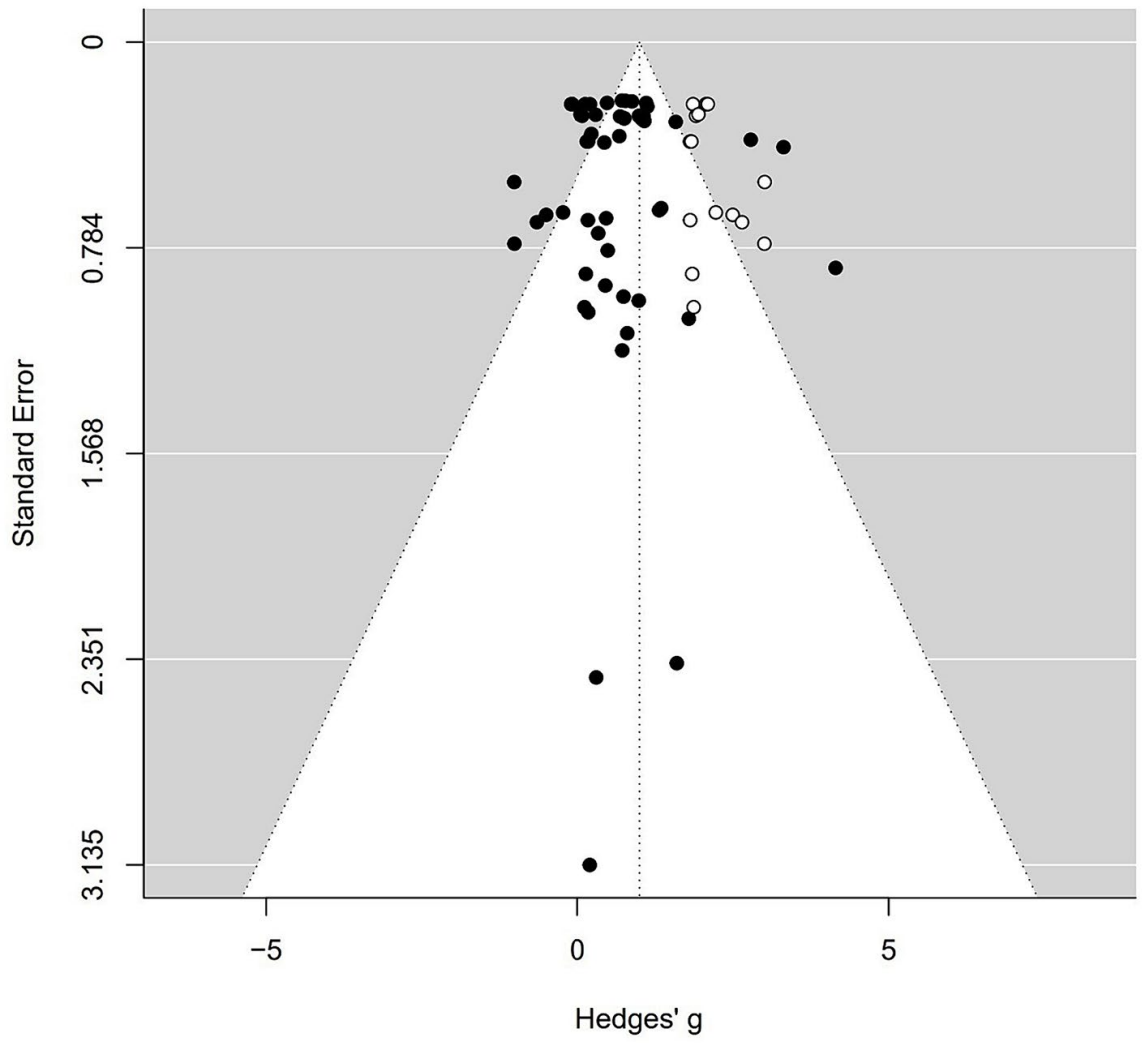
Funnel plot of standard error by Hedges g.

To assess the potential presence of publication bias in our meta-analysis of team-building intervention on cohesion, we utilized a funnel plot for visual examination, as illustrated in Figure 4. In an ideal scenario without publication bias, data points (depicted as solid circles) from individual case studies would exhibit a symmetrical distribution. Any deviation from this symmetry suggests the potential presence of publication bias. As seen in Figure 4, the distribution of effect sizes is slightly left–skewed.

Applying the trim-and-fill method by Duval and Tweedie (2000) reveals that 15 missing studies on the right side are required to achieve symmetry in the funnel plot. The required 15 additional cases are shown on the right as hollow circles in Figure 4.

We also assessed publication bias using Rosenthal’s (1979) fail-safe N (N*_fs_*) concept. When N*_fs_* exceeds 5 k + 10, where k represents the number of included case studies, it is unlikely to substantially impact the average effect size. In our specific study, with k equal to 52, the meta-analysis results remain stable as long as the N*_fs_* exceeds 270. Our N*_fs_* value is 2,570, well above the 270 threshold, emphasizing the robustness of the meta-analysis. In simpler terms, even if more than 2,570 studies with zero effect size were introduced, the overall results would remain largely unaltered.

According to the trim-and-fill method by Duval and Tweedie (2000), an adjusted effect size of 1.00 (95% CI = [0.75; 1.25]), larger than the calculated effect size of 0.65, is presented.

### 3.4 Type of cohesion measure

Table 1 presents 52 effect sizes calculated for four cohesion types (GI-T, GI-S, ATG-T, and ATG-S). Notably, task cohesion exhibited a larger effect size than social cohesion. ATG-T showed a significant large effect size (ES = 1.06, 95% CI = [0.17; 1.95]). The other three cohesion types, GI-T (ES = 0.56, 95% CI = [0.23; 0.89]), ATG-S (ES = 0.56, 95% CI = [0.22; 0.91]), and GI-S (ES = 0.52, 95% CI = [0.01; 1.02]), showed a moderate effect size. According to meta-ANOVA, the differences between the four cohesion types were not statistically significant (*F*(3, 48) = 1.312, *p* > 0.05).

### 3.5 Moderator variables

This study examined the effectiveness of team-building concerning five different moderators. These moderators encompassed the effectiveness of team-building on cohesion across gender, age, sample size, intervention duration, and athletes’ skill level. Notably, the only significant moderator identified was athletes’ skill level. No statistically significant differences were observed within the other four moderators (refer to Table 2 for details).

**Table 2:** Moderator effects.

Potential moderator	*F*-test	*p*	Effect size	*n*	95% CI
Average effect size from individual studies			0.65	52	[0.42; 0.89]
Gender	*F*(1, 50) = −0.169	ns			
Female only			0.63	14	[0.43; 0.84]
Male only			0.66	38	[0.32; 1.00]
Mean age	*F*(2, 49) = 2.306	ns			
<15 years			0.48	8	[0.17; 0.78]
15–20 years			0.88	29	[0.60; 1.15]
>20 years			0.25	15	[−0.45; 0.96]
Sample size	*F*(2, 49) = 0.304	ns			
<20			0.64	17	[0.49; 1.21]
20–30			0.85	18	[0.49; 1.21]
>30			0.50	17	[−0.03; 1.31]
Length of intervention	*F*(2, 49) = 0.264	ns			
Less than 2 weeks			0.31	2	[−0.97; 1.60]
2 to 20 weeks			0.69	32	[0.31; 1.06]
20 weeks and above			0.62	18	[0.43; 0.82]
Skill level	*F*(2, 49) = 3.315	<0.05			
High School			0.77	16	[0.59; 0.95]
Intercollegiate			1.13	16	[0.53; 1.72]
Professional club			0.40	20	[−0.02; 0.83]

#### 3.5.1 Gender

As indicated in Table 2, there is a slightly larger effect size for male athlete teams (ES = 0.66, 95% CI = [0.32; 1.00]) compared to female athlete teams (ES = 0.63, 95% CI = [0.43; 0.84]). However, the difference is not statistically significant (*p* > 0.05).

#### 3.5.2 Age

We categorized the ages of the participants into three groups. In the 15–20 years old category, we observed a significant large effect size (ES = 0.88, 95% CI = [0.60; 1.15]), while those under the age of 15 showed a significant moderate effect size (ES = 0.48, 95% CI = [0.17; 0.78]). However, the effect size (ES = 0.25, 95% CI = [−0.45; 0.96]) for those over the age of 20 was not statistically significant. The meta-ANOVA analysis indicated that the difference between these three categories was not statistically significant (*p* > 0.05). Consequently, age was not identified as a significant moderator in this study.

#### 3.5.3 Sample size

The sample size was divided into three groups. In the category with 20–30 participants, we observed a significant large effect size (ES = 0.85, 95% CI = [0.49; 1.21]). Additionally, the category with under 20 participants showed a significant moderate effect size (ES = 0.64, 95% CI = [0.49; 1.21]). However, the effect size (ES = 0.50, 95% CI = [−0.03; 1.31]) for those over 30 participants was not statistically significant. The meta-ANOVA analysis indicated that the difference between these three categories was not statistically significant (*p* > 0.05). Consequently, the sample size was not identified as a significant moderator in this study.

#### 3.5.4 Length of intervention

The team-building interventions in our study varied in duration, ranging from 1 day to the entire sports season. As shown in Table 2, a significant moderate effect size (ES = 0.69, 95% CI = [0.31; 1.06]) was observed for interventions lasting between 2 and 20 weeks. Additionally, a significant moderate effect size was evident for interventions extending for 20 weeks or longer (ES = 0.62, 95% CI = [0.43; 0.82]). However, the effect size (ES = 0.31, 95% CI = [−0.97; 1.60]) for intervention durations less than 2 weeks was not statistically significant. The meta-ANOVA analysis indicated that the difference between these three categories was not statistically significant (*p* > 0.05). Consequently, the length of intervention was not identified as a significant moderator in this study.

#### 3.5.5 Skill level of the athletes

As outlined in Table 2, we observed a significant large effect size (ES = 1.13, 95% CI = [0.53; 1.72]) in the category of collegiate teams, while we identified a significant moderate effect size (ES = 0.77, 95% CI = [0.59; 0.95]) in the category of high school teams. However, the effect size (ES = 0.40, 95% CI = [−0.02; 0.83]) was not statistically significant for professional teams.

According to the meta ANOVA and post-hoc test results, significant differences (*p* < 0.05) in the effectiveness of team-building on cohesion were found between collegiate teams and professional teams. Consequently, athletes’ skill level can act as a moderator in the effectiveness of team-building intervention on cohesion.

### 3.6 Meta-regression analysis

**Figure 5:**
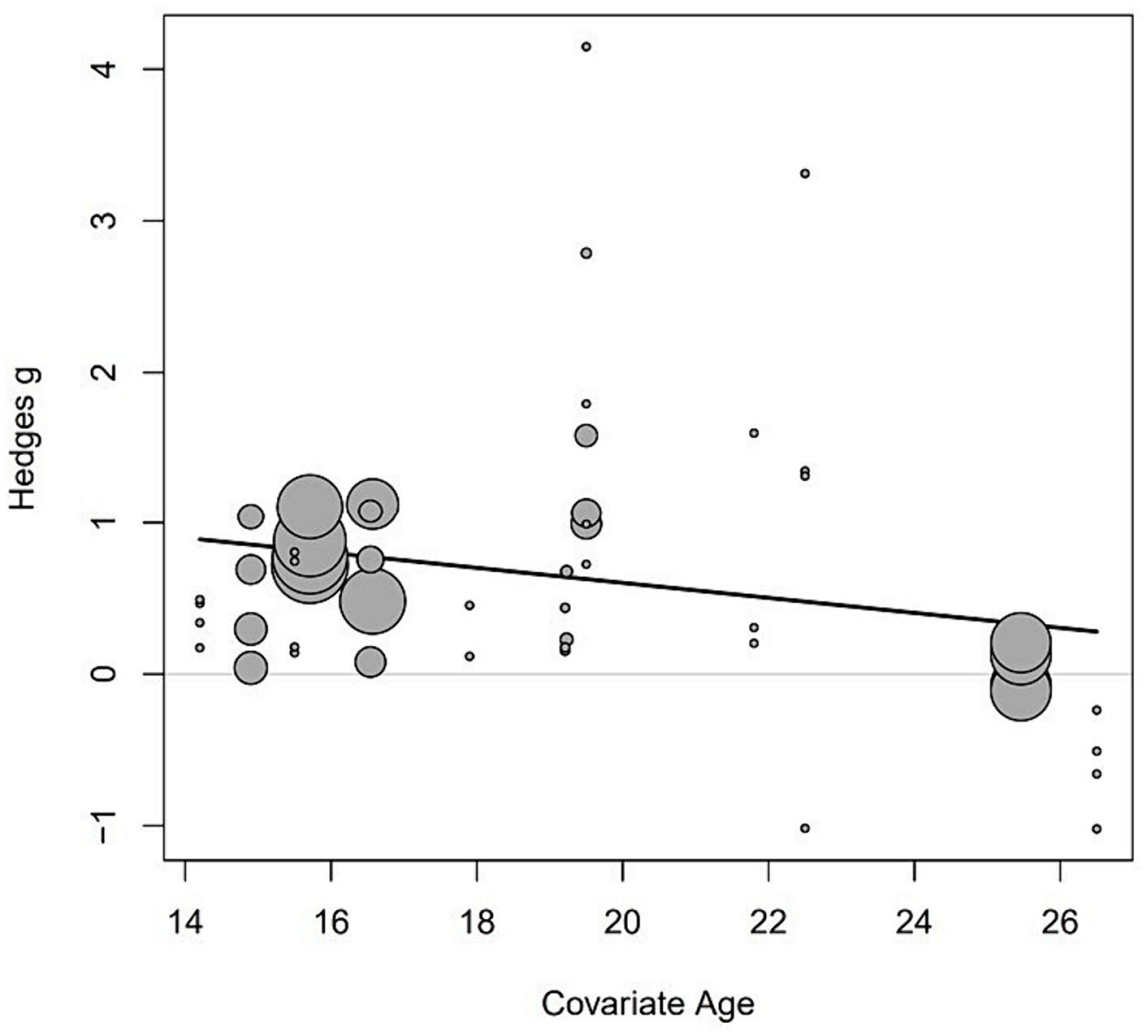
Meta-regression analysis of the relationship between Hedges’ g and the mean age of participants.

We conducted meta-regression analyses to explore the association between three independent variables (age, sample size, and duration in weeks) and the effect size. The results of meta-regression analysis showed that the effect size tend to decrease with mean age, although this association did not reach statistical significance (*p* > 0.05) (refer to Figure 5 and Table 3). Furthermore, the relationships between sample size and effect sizes, as well as the relationship between duration in weeks and effect sizes, did not show statistical significance (*p* > 0.05) (refer to Table 3).

**Table 3:** Univariate meta-regression analysis.

Moderator	*β*	SE	*t*	*p*
Mean age	−0.044	0.033	−1.339	0.187
Sample size	−0.004	0.010	−0.351	0.727
Duration (in weeks)	−0.012	0.022	−0.549	0.587

## 4 Discussion

The main goal of this meta-analysis is to assess the impact of team-building interventions on cohesion, a critical element in sports teams that plays a pivotal role in task execution and fostering social interactions (Carron and Spink, 1993; Carron et al., 1997). If team-building interventions focused on fostering cohesion can establish a sense of unity among team members, they have the potential to serve as catalysts for enhancing overall team performance.

Our study’s key finding is that team-building activities indeed improve cohesion in sports teams. Among various measures of cohesion, we found that team-building interventions were most successful in enhancing ATG-T, followed by GI-T, GI-S, and ATG-S. Some team-building activities focus on social aspects, like team camping trips (Cogan and Petrie, 1995), ropes and challenge courses (e.g., Meyer, 2000), and informal social gatherings (e.g., Yukelson, 1997). These activities are likely to enhance social cohesion within the team. On the other hand, other team-building activities concentrated on team goals and tasks, such as team goal-setting (e.g., Senécal et al., 2008; Kim et al., 2017; Durdubas and Koruc, 2023), tasks relevant to team performance (e.g., Leo et al., 2021), clarifying roles (e.g., Tassi et al., 2023), and adhering to team norms (e.g., Prapavessis et al., 1996). These activities are expected to primarily improve task cohesion within the team. Notably, our analysis revealed a stronger impact of team-building activities on task cohesion compared to social cohesion due to the predominant focus on tasks and objectives rather than social interactions in the studies examined.

Another aim of our study is to explore how various moderator variables affect the improvement of cohesion through team-building intervention. Several findings are associated with the influence of moderators. To begin with, we explored gender as a potential moderator. The findings indicate that team-building interventions are equally effective for teams composed solely of females as well as those with only males. In our meta-analysis, using gender served as a potential moderator, the results of the meta *t*-test showed no significant difference (*p* > 0.05) in the effectiveness of team-building interventions applied to both men’s and women’s teams. This aligns with the results reported by Martin et al. (2009).

In this study, the second potential modulator under scrutiny was the age of participants. We categorized subjects of individual study into three age groups, and then the effect size was calculated with age as a moderate variable. We found that the age category of 15–20 exhibited a large effect size, while the category under 15 years old showed a significant moderate effect size. However, there was no significant effect size observed for the category of those aged over 20 years. Consequently, we can conclude that team-building is most effective for sports teams with members between 15 and 20 years old, while it does not show effectiveness for sports teams with members aged over 20.

In our analysis, the third potential modulator we explored was sample size. We classified the sample size of each study into three groups, and then the effect size was calculated with sample size as a moderate variable. In the group with 20–30 participants, a significant large effect size was observed, while the category with under 20 participants showed a significant moderate effect size. However, there was no significant effect size observed for the category of those with over 30 participants. As a result, we can conclude that team-building is most effective for sports teams ranging from 20 to 30 members, while it does not show effectiveness for sports groups with over 30 members.

In our analysis, the fourth potential modulator we explored was athletes’ skill level, which turned out to be the only significant moderator in this study. Team-building interventions were most effective for collegiate teams, followed by high school teams, while the effectiveness in professional teams did not reach statistical significance (*p* > 0.05). This discrepancy may be explained by a potential ceiling effect, given that professional athletes typically possess a strong understanding of cohesion. Consequently, while professional teams do benefit from team-building interventions, the extent of improvement may be comparatively modest due to their already robust cohesion and training. The meta-ANOVA indicated that the differences between the three groups were statistically significant (*p* < 0.05), and the post-hoc test revealed that the effect size of the collegiate team was larger than that of the professional club team. Thus, it can be concluded that team-building is most effective for collegiate sports teams, while it does not show effectiveness for professional club teams.

Moving on to the fifth potential modulator, we explored intervention duration. The articles in this meta-analysis encompassed team-building interventions with durations ranging from a single day to an entire sports season. Notably, interventions lasting less than 2 weeks did not yield noticeable improvements in cohesion and were not statistically significant, aligning with the findings of Martin et al. (2009). Conversely, Shipherd et al. (2014) conducted a single-day team-building intervention with a collegiate rugby team and observed a significant increase in team cohesion. These disparities in intervention duration underscore the need for meta-analytic investigations to gain a comprehensive understanding of the optimal duration required for team-building interventions to enhance cohesion in future studies.

Although numerous studies have demonstrated the positive effects of team-building interventions on cohesion, there are instances, as seen in some studies (Prapavessis et al., 1996; Kwon, 2022), where significant improvements were not observed. The intervention period might have impacted why there wasn’t a significant change in group cohesion after the team-building program was implemented. Kwon (2022) and Prapavessis et al. (1996) conducted a team-building intervention over 8 weeks but did not find a clear improvement in group cohesion. This suggests that the intervention duration might have been too short to see significant differences in these studies. Group cohesion improves gradually through changing members’ perceptions and resolving conflicts that arise during interactions. Therefore, steady progress over a long enough time is important. However, conducting long-term team-building interventions can be challenging due to various environmental factors.

## 5 Conclusion

In conclusion, this study provides several key insights into the impact of team-building intervention on cohesion within sports teams. Firstly, team-building activities predominantly enhance task cohesion rather than social cohesion within sports teams. Different approaches to team-building, focusing on either social interactions or team goals and tasks, result in corresponding improvements in cohesion. Thus, social cohesion benefits from team-building activities emphasizing social interaction, while task cohesion improves when activities concentrate on team objectives.

Secondly, team-building interventions are most effective for individuals aged 15–20 and within collegiate sports teams. Conversely, the expected positive effects may not be noticeable when subjects are over 20 years old and belong to professional league teams.

Thirdly, interventions lasting longer than 2 weeks are crucial for enhancing team cohesion. Conversely, the expected positive effects may not be noticeable if the intervention period is less than 2 weeks. Based on our findings, an intervention period of at least 2 weeks is necessary to see the effects of a team-building intervention on group cohesion in sports teams. However, it is not necessarily the case that a longer intervention period will result in a greater intervention effect. Additionally, the time delay of the intervention was not investigated in this study. Therefore, the association between the team-building intervention period and group cohesion remains unclear. Further research is needed to determine the optimal intervention period that significantly affects group cohesion. It is also important to consider the time delay of intervention. Furthermore, there is possibility that a group cohesion may be influenced by multiple processes rather than just team-building alone. Therefore, claiming that team-building alone enhances group cohesion may not be reasonable. Therefore, decision-makers in sports teams should carefully consider the duration and realistic expectations of team-building interventions. In any case, to have an effective team-building intervention, it is necessary to implement the intervention for a long enough period. To address this, leaders should ensure interventions are implemented over a sufficient period to yield meaningful results.

In summary, team-building interventions can significantly enhance cohesion within sports teams, particularly when tailored to specific team dynamics and implemented over a sufficient duration.

Nevertheless, it’s important to note the limitations of this meta-analysis. First and foremost, the study focused exclusively on interactive sports, suggesting the need for future research to explore and compare the effectiveness of team-building interventions in both interactive and coactive sports settings. Secondly, the review concentrated solely on immediate post-intervention effects, emphasizing the necessity for longitudinal studies to gain a more profound understanding of the lasting benefits of team-building interventions for sports teams over an extended period.

## Data availability statement

The raw data supporting the conclusions of this article will be made available by the author, without undue reservation.

## Author contributions

SK: Data curation, Formal analysis, Investigation, Methodology, Validation, Visualization, Writing – original draft, Writing – review & editing.

## Footnotes

^1^ r-project.org
